# Function Electrical Stimulation Effect on Muscle Fatigue Based on Fatigue Characteristic Curves of Dumbbell Weightlifting Training

**DOI:** 10.34133/cbsystems.0124

**Published:** 2024-06-06

**Authors:** Shihao Sun, Guizhi Xu, Mengfan Li, Mingyu Zhang, Yuxin Zhang, Wentao Liu, Alan Wang

**Affiliations:** ^1^ State Key Laboratory of Reliability and Intelligence of Electrical Equipment, School of Electrical Engineering, Hebei University of Technology, 300132 Tianjin, China.; ^2^ Hebei Key Laboratory of Bioelectromagnetics and Neuroengineering, 300132 Tianjin, China.; ^3^ Tianjin Key Laboratory of Bioelectromagnetic Technology and Intelligent Health, Hebei University of Technology, 300132 Tianjin, China.; ^4^ School of Electrical Engineering, Hebei University of Technology, 300132 Tianjin, China.; ^5^ School of Health Sciences and Biomedical Engineering, Hebei University of Technology, 300132 Tianjin, China.; ^6^ Auckland Bioengineering Institute, University of Auckland, Auckland, New Zealand.; ^7^ Centre for Brain Research, Faculty of Medical and Health Sciences, University of Auckland, Auckland, New Zealand.; ^8^ Centre for Medical Imaging, Faculty of Medical and Health Sciences, University of Auckland, Auckland, New Zealand.

## Abstract

The parameter setting of functional electrical stimulation (FES) is important for active recovery training since it affects muscle health. Among the FES parameters, current amplitude is the most influential factor. To explore the FES effect on the maximum stimulation time, this study establishes a curve between FES current amplitude and the maximum stimulation time based on muscle fatigue. We collect 10 subjects’ surface electromyography under dumbbell weightlifting training and analyze the muscle fatigue state by calculating the root mean square (RMS) of power. By analyzing signal RMS, the fatigue characteristic curves under different fatigue levels are obtained. According to the muscle response under FES, the relationship curve between the current amplitude and the maximum stimulation time is established and FES parameters’ effect on the maximum stimulation time is obtained. The linear curve provides a reference for FES parameter setting, which can help to set stimulation time safely, thus preventing the muscles from entering an excessive fatigue state and becoming more active to muscle recovery training.

## Introduction

At present, stroke has become one of the most serious neurological diseases, which is usually accompanied by movement disorders and cognitive impairment [1]. In recent years, the number of stroke patients has increased annually [2]. Most stroke patients are accompanied by movement disorders, which seriously affect the normal life of patients. Functional electrical stimulation (FES) technology is a new type of treatment, which is through the simulation of the nerve on the muscle issued by the electrical signals for rehabilitation training [2,3].

FES can promote blood circulation, prevent muscle atrophy, and help patients restore limb motor function, so the technology has been widely studied [4]. Nowadays, the use of FES technology has been carried out in human safety, and for parameter setting, the fixed parameter method is mostly used [1,5,6]. In the study of muscle fatigue induced by low-frequency FES, Wang et al. [7] verified that low-frequency FES is more suitable for measuring muscle fatigue changes by comparing the fatigue induced by spontaneous exercise and FES. Zhou et al. used 6 stimulation schemes to carry out FES experiments according to the division of pulse width and frequency in the influence of different neuromuscular FES on muscles. Finally, it was proved that the stimulation frequency had the greatest influence on muscle fatigue speed, and the effect of asynchronous stimulation was better than that of synchronous stimulation [8]. Li et al. pointed out in the study of FES motion modeling and control based on neural network that the setting of stimulation parameters is mostly determined by empirical values and is mainly based on fixed parameters. The neural network model he established is also suitable for elbow joint motion under FES [9]. In the design of the master–slave FES rehabilitation system, Yao et al. [10] successfully used FES technology to drive patients to carry out wrist rehabilitation training, which laid a foundation for the development of master–slave FES technology. Although they have made some key achievements in the study of FES technology, in their research, FES parameters are relatively fixed. When the FES parameters are changed, whether the conclusion changes is also unknown [6,11,12]. Their study did not point out the effect of FES on muscle fatigue. Therefore, in order to make full use of FES technology, it is necessary to explore the influence of parameter changes of FES on muscles, so that the use of FES technology does not cause damage to muscles caused by muscle fatigue, to better use electrical stimulation technology, and then provide a better recovery effect.

In exploring the influence of parameter changes on muscles, it is necessary to pay attention to the 2-sidedness of FES technology [13]. Studies have shown that FES technology can alleviate muscle fatigue, and its mechanism is similar to physical therapy massage [14]. However, excessive use of FES will cause muscle fatigue, which will cause muscle damage. Therefore, it is very important to explore the relationship between the stimulation time and the setting of stimulation parameters, and to find the maximum stimulation duration. In the current research on muscle fatigue, the classification method is used to divide the fatigue state mainly into 3 classifications and 5 classifications [15–17], as shown in Table. In general, peripheral muscle fatigue is caused by the energy consumption of the muscles, so the fatigue state of the muscles can be determined by analyzing the energy changes of the muscles, and the energy changes of the muscles can be obtained by collecting the subjects’ surface electromyography (sEMG) [18]. We also use the classification of sEMG to determine muscle fatigue stage, and then provide a reference for FES parameter setting [19].

**Table.** Common fatigue division state

	Fatigue grading				
Engineering classification	Non-fatigue		Fatigue		
	Non-fatigue	Defatigation		Fatigue	
Muscle isometric contraction classification	Non-fatigue	Loading weight	Defatigation	Deep fatigue	Fatigue

In this paper, a curve of FES current amplitude and stimulation time based on the fatigue characteristic curve is proposed. Based on the characteristics of muscle fatigue under dumbbell weightlifting training, this paper establishes the relationship curve between the FES current amplitude and the stimulation time, explores other parameters’ effect on the stimulation time, and provides a method for adjusting parameters and avoiding fatigue damage for the use of FES technology. Specifically, the experiment obtained 10 subjects’ sEMG signals under dumbbell weightlifting training. By analyzing sEMG-RMS (root mean square), the muscle fatigue characteristic curves under different muscle fatigue levels are obtained. Then, according to the muscle response under FES, the relationship curve between the maximum stimulation time and the current amplitude is obtained. The curve shows that there is a certain linear relationship between the FES current amplitude and the stimulation time, and the linear relationship is also verified in the cross-subject experiment. The finding of this relationship avoids muscle damage caused by a very long stimulation time or an unreasonable parameter setting, and lays a foundation for the better use of FES technology.

## Materials and Methods

### Participants

Ten healthy volunteers, 9 males and 1 female, aged 23 ± 3 years, 170 ± 5 cm in height, and 130 ± 5 kg in weight (105 kg for the female), were selected for the experiment. The content of the experiment was informed before the experiment and the consent was obtained. Three days before the experiment, there was no disease, no medication, and no muscle damage at the experimental site.

### Equipment

The equipment needed for the experiment were dumbbells, FES equipment, and sEMG equipment. Among them, dumbbells of 5-kg, 10-kg, 15-kg, and 20-kg standard weights were used for dumbbell weightlifting training. The FES device adopted an integrated adjustable parameter electrical stimulation device, which can adjust the size, frequency, and time history of the output current. In the experiment, the control variable method was used to explore the relationship between FES parameters and the stimulation time. The sEMG equipment was selected from Neuracle company, and the sampling frequency is 1,000 Hz.

### Experimental design

**Fig. 1.**
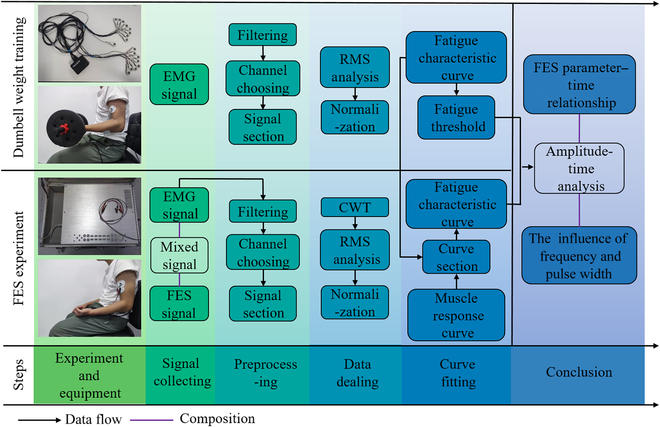
Experimental flowchart. The picture shows the arm’s position and equipment of dumbbell weightlifting training and FES experiment.

The experiments were divided into dumbbell weightlifting training and FES experiment. The experimental process is shown in Fig. 1. The ultimate goal of dumbbell weightlifting training was to obtain subjects’ fatigue characteristic curves at different fatigue levels and determine the fatigue threshold. The purpose of the FES experiment was to explore the FES parameter effect on muscle fatigue.

#### Dumbbell weightlifting training

Dumbbell weightlifting training was used to obtain a healthy body’s fatigue characteristic curve. Each subject needed 3 experiments, and each group of experiments ensured that the subjects were in a non-fatigue state at first.

During the experiment, the subjects needed to lift the dumbbell of the same weight (10 kg for men and 5 kg for women) with the arm’s power; the right hand’s palm was facing upward, the upper arm was parallel to the body, and the upper arm and the forearm were kept perpendicular to the experimental state. The collected sEMG began with the subjects entering the experimental state, and during the experiment, encouragement was needed to be given to the subjects and it was ensured that collection time was greater than 1 min. The experiment ended when the subjects ' arms cannot remain vertical or the subjects’ muscle had a strong subjective fatigue consciousness.

#### FES experiment

The FES experiment is used to determine subjects’ fatigue generation time under different current amplitudes and the effect of changing the current frequency and amplitude on the fatigue generation time. First of all, it was ensured that the subjects were in a non-fatigue state, the forearm was placed steadily on the leg, and the upper arm was kept vertical.

**Fig. 2.**
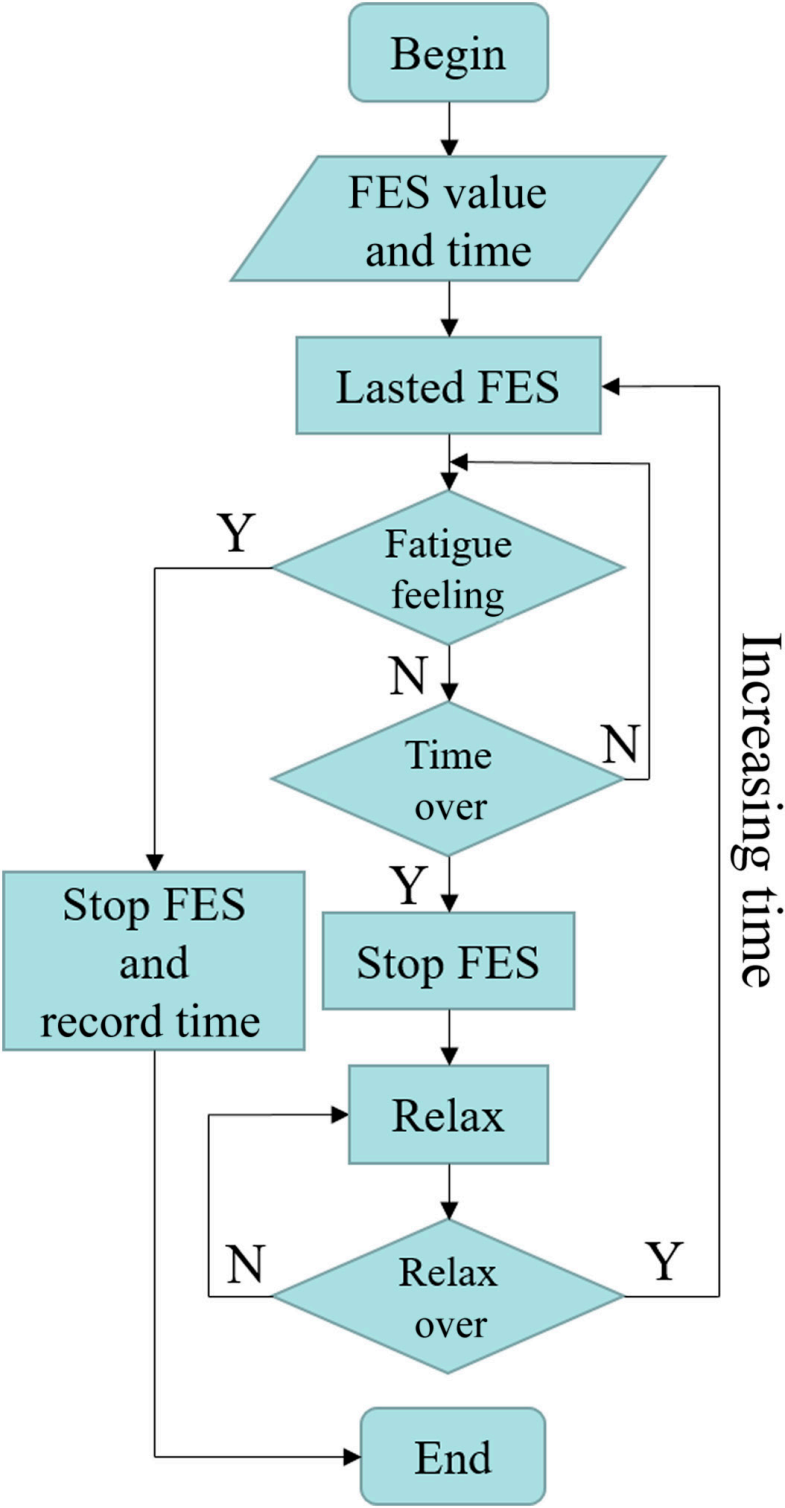
Flowchart of the determination method of electrical stimulation time.

In exploring the influence of changing current amplitude on the maximum stimulation time, the initial FES parameters were set according to the empirical value with a frequency of 24 Hz and a pulse width of 200 μs [20,21]. In the experiment, the current range is 2 to 15 mA, and each 1-mA increase in the current is a set of experiments, and other parameters remained unchanged. Each set of experiments carried out several exploratory experiments until the accurate muscle response curve under FES was determined. The experimental design is shown in Fig. 2. It was necessary to determine the most appropriate stimulation time according to the subjects’ subjective feelings, and the subjects’ sEMG during the FES experiment was obtained [22].

For the effect of frequency and pulse width on the maximum stimulation time, the current amplitude was kept at 12 mA, the current frequency was increased or the current pulse width was increased, and other conditions were consistent with the current amplitude.

### Data collection and processing method

**Fig. 3.**
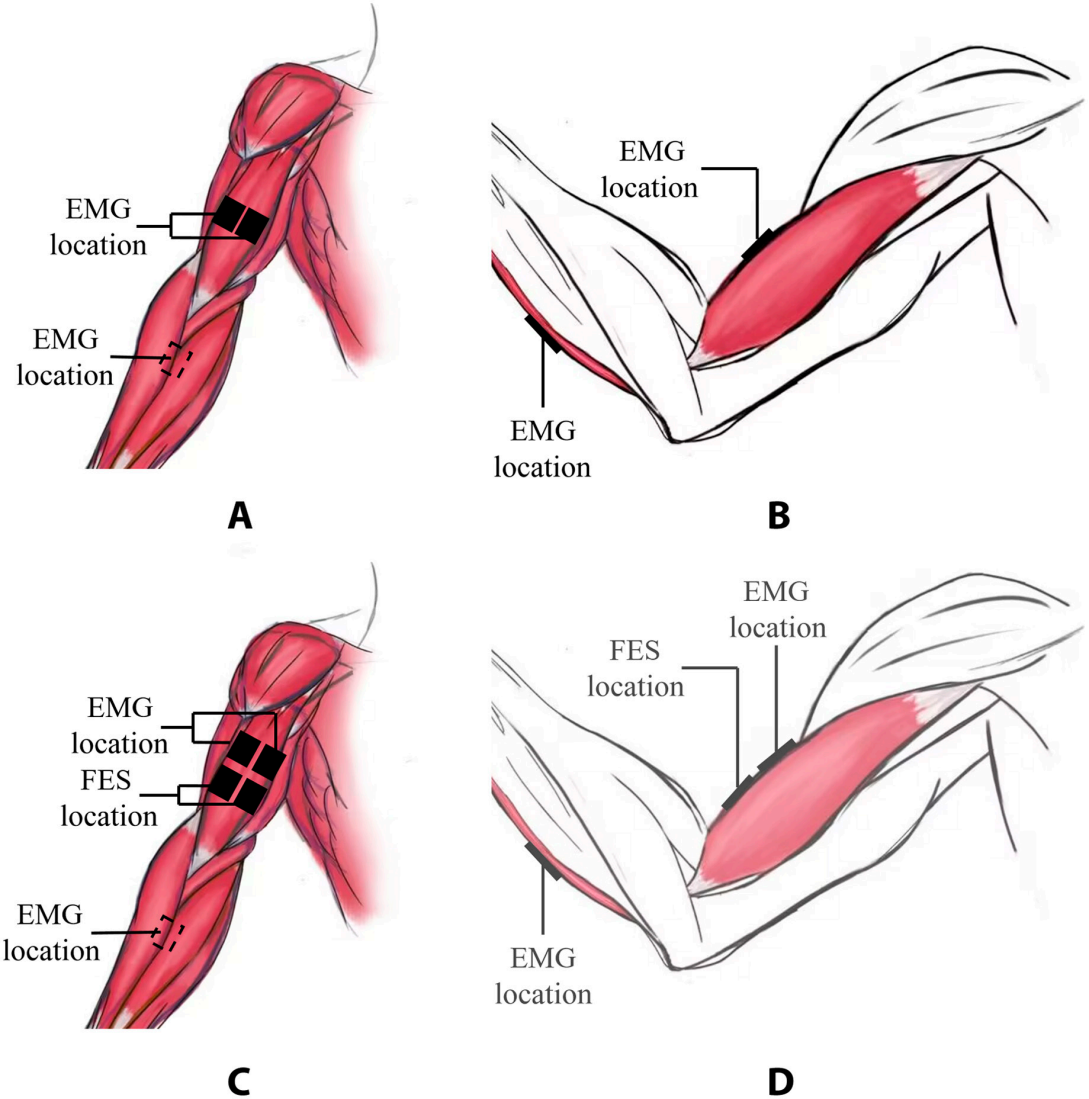
Electrode patch position. (A) The position of the front electrode patch of the dumbbell weightlifting training arm, where the dotted line represents the back position of the arm. (B) Dumbbell weightlifting training arm side. (C) FES training arm front electrode patch position, where the dotted line represents the back of the arm position. (D) FES training arm side.

The position of the electrode patch required for the dumbbell weightlifting training and electrical stimulation experiment is shown in Fig. 3. The sEMG collected all came from the biceps brachii. Before the experiment, it was necessary to use cotton with alcohol to remove the oil on the skin surface [23]. For the sEMG obtained from the experiment, it was necessary to use the Butterworth band-pass filter for filtering processing, and the passband range is 20 to 450 Hz [24,25].

#### Wavelet transform

**Fig. 4.**
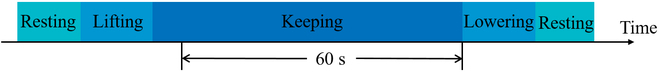
Stage diagram of sEMG collected in dumbbell weightlifting training.

In the process of collecting sEMG during dumbbell weightlifting training, as shown in Fig. 4, the part of 60 s before the end of the experiment was taken for further analysis [22]. The signals collected during the FES experiment were a mixed signal; thus, it was necessary to separate the signal and extract the muscle response signal in the mixed signal. The method used in this paper is wavelet transform. The formula is as follows:

$$\begin{array}{c} \text{WT}\left(a,\tau \right)=\frac{1}{\sqrt{a}}\int_{-\infty }^{\infty }f\left(t\right)*\psi \left(\frac{t-\tau }{a}\right)\mathrm{dt} \end{array}$$ (1)

Here, *a* is the transform scale, τ is the translation, and *f*(*t*) is the original mixed signal. In this paper, *a* is 128 [16], and the response signal of the muscle under FES can be obtained, in other words, the original sEMG under FES.

#### RMS and normalization

For the sEMG collected by the experiment, the distance of each window movement was set to 1,000 sampling points, and the time domain feature RMS of the muscle in each time window was calculated [26]. The formula is as follows:

$$\text{RMS}=\sqrt{\frac{x_{1}^{2}+x_{2}^{2}+x_{3}^{2}+\cdots +x_{n}^{2}}{n}}$$ (2)

Here, *n* is the number of sampling points. In this paper, *n* = 1,000, and *x*_1_, *x*_2_, *x*_3_, …*x_n_* are the original different subjects’ sEMG. The subjects’ RMS characteristic curves can be determined.

In order to eliminate the influence of different subjects’ muscle strength differences, it was necessary to normalize all datasets. The method used in this paper is max–min standardization [27]. The formula is as follows:

$$r\prime =\frac{r-A_{\min}}{A_{\max}-A_{\min}}$$ (3)

Among them, *A_max_* and *A_min_* are the maximum and minimum values in the RMS characteristic curve, and *r* is each RMS value in the RMS characteristic curve. In this way, the RMS characteristic curve can be uniformly mapped to the interval [0, 1].

#### Function fitting

The fatigue characteristic curves under different fatigue levels were obtained by fitting the RMS characteristic curve. Among them, the RMS characteristic curves under dumbbell weightlifting training were fitted by power function, and the FES experiment was fitted by polynomial function. The fitting results were represented by iRMS to obtain the fatigue characteristics of muscles under different fatigue levels [28].

The fatigue characteristic curve under dumbbell weightlifting training reflected the energy change trend of sEMG when the subjects were fatigued. The fatigue characteristic curves under the FES experiment reflected the change trend of muscle energy caused by electrical signals, and the final result of the 2 energy changes was to cause the muscle to enter the fatigue state. Therefore, by comparing the energy changes of sEMG under the 2 experiments, the muscle fatigue time caused by FES can be determined, in other words, the maximum stimulation time.

By changing the output current frequency and pulse width, the influence of frequency and pulse width on the maximum stimulation time can be obtained. By obtaining the maximum stimulation time under different current amplitudes, the mapping relationship between the maximum stimulation time and the current amplitude can be established, and then the relationship curve can be determined. Finally, the relationship curve was subjected to a cross-subject experiment to verify the accuracy of the experimental results.

## Results

### Muscle fatigue analysis

**Fig. 5.**
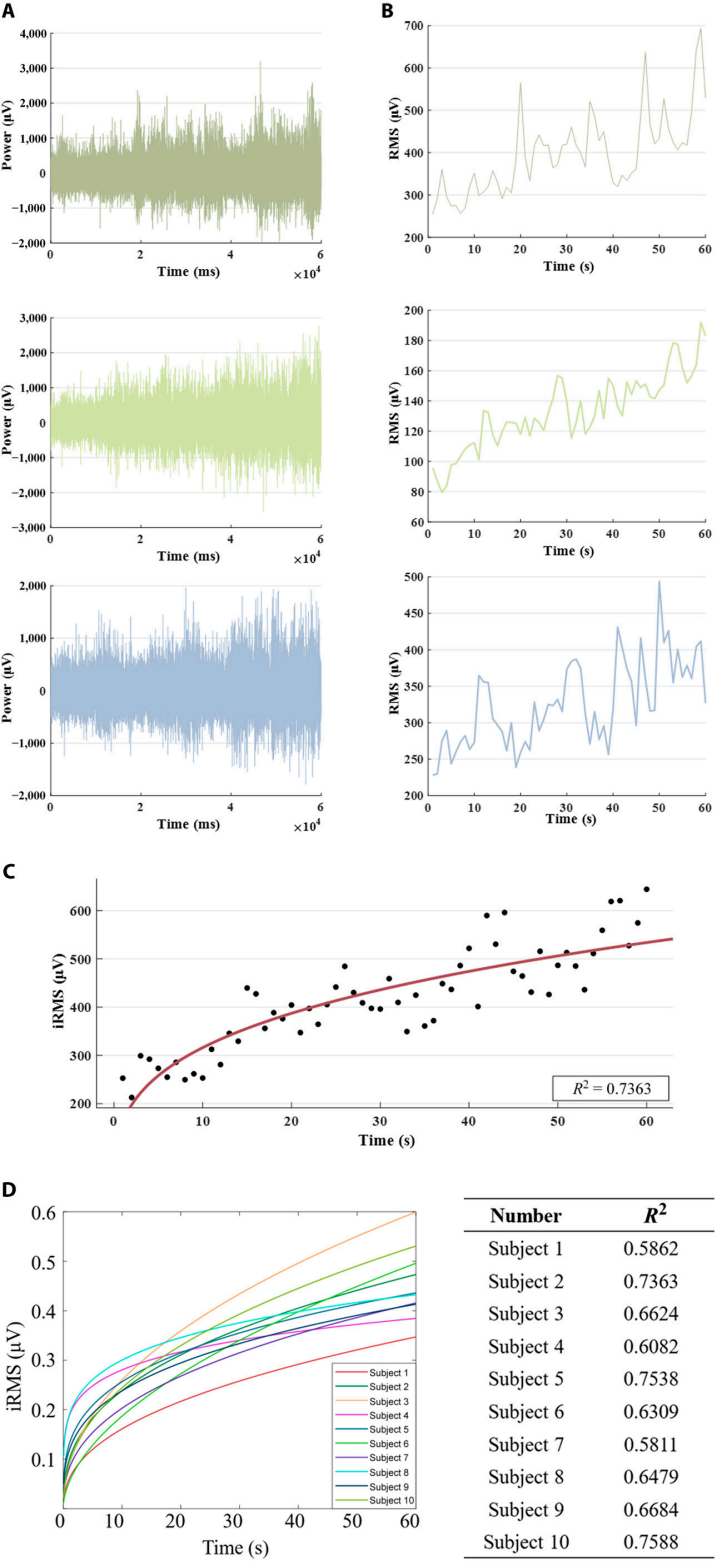
sEMG data under dumbbell weightlifting training. (A) Original sEMG. (B) RMS characteristic curve (C) The power function fitting results of the normalized RMS characteristic curve. (D) Ten healthy subjects’ RMS curve fitting results.

In order to explore the fatigue changes of the muscles under the training of dumbbell weightlifting, Fig. 5A shows subjects’ sEMG under dumbbell weightlifting training. It can be seen from the figure that the amplitude of the sEMG increases obviously with the training under the weight-bearing state, and for different subjects, the range of amplitude increase also changed. Each subject’s RMS characteristic curve was made, and it can be seen that the subject’s RMS values also increased with the training. The results of power function fitting of the RMS characteristic curve are shown in Fig. 5C. The fitting curve can well reflect the fatigue characteristic curve of muscle. All subjects’ fatigue characteristic curves were made, and the fitting curves were statistically significant (*P* < 0.05), indicating that the characteristics can be expressed by power function fitting.

The fatigue characteristic curves of different subjects were normalized, and the results are shown in Fig. 1D; it can be seen from the figure that different subjects’ fatigue characteristic curves were consistent. When the subjects entered the fatigue state, the difference of the maximum threshold was caused by the difference of the subjects’ own muscle strength [29]. Although the normalization process cannot completely eliminate this difference, it can greatly weaken this difference. The curve showed the change of muscle fatigue state.

### Muscle response to FES

**Fig. 6.**
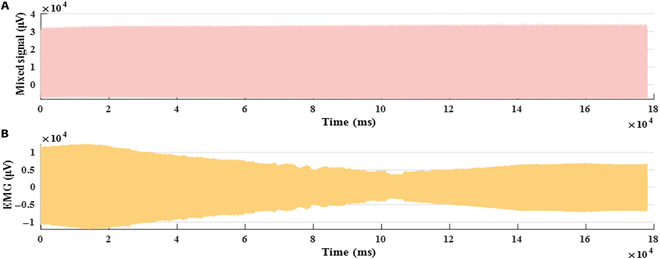
Comparison diagram of signal separation. (A) Mixed signal before separation. (B) Evoked sEMG after separation.

**Fig. 7.**
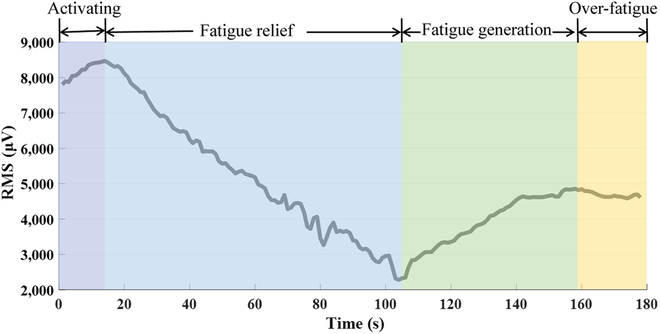
Muscle fatigue response curve and fitting results under FES.

The sEMG response of the muscle under FES is shown in Fig. 6A. The signals contained the electrical signal output by the FES device and the FES-induced sEMG. The sEMG signals separated by the continuous wavelet transform (CWT)are shown in Fig. 6B. The signals are the original sEMG of the muscle under FES. The graph shows the change of muscle response to electrical signal over time under FES. The time domain analysis of the signal was carried out to obtain the response of the muscle under FES. The result is shown in Fig. 7. We found that the curve is “M” wave [10]; thus, the curve can be divided into 4 sections:

1. Muscle activation characteristic stage: when the muscle is stimulated by external electrical signals, it changes from a resting state to an active state [30].

2. Muscle recovery characteristic stage: muscle factor starts to absorb energy from a more active state and becomes gradually stable.

3. Muscle fatigue characteristic stage: muscle factor began to release energy to resist the influence of electrical signals on the muscle. At this stage, the subjects had obvious muscle soreness, indicating that the muscle is entering the fatigue state.

4. Excessive fatigue stage: After entering this state, the energy of muscle factor is not enough to offset the influence of electrical signals, and muscle activity decreases. From this stage, if electrical stimulation continues, it will cause inevitable damage to muscles.

**Fig. 8.**
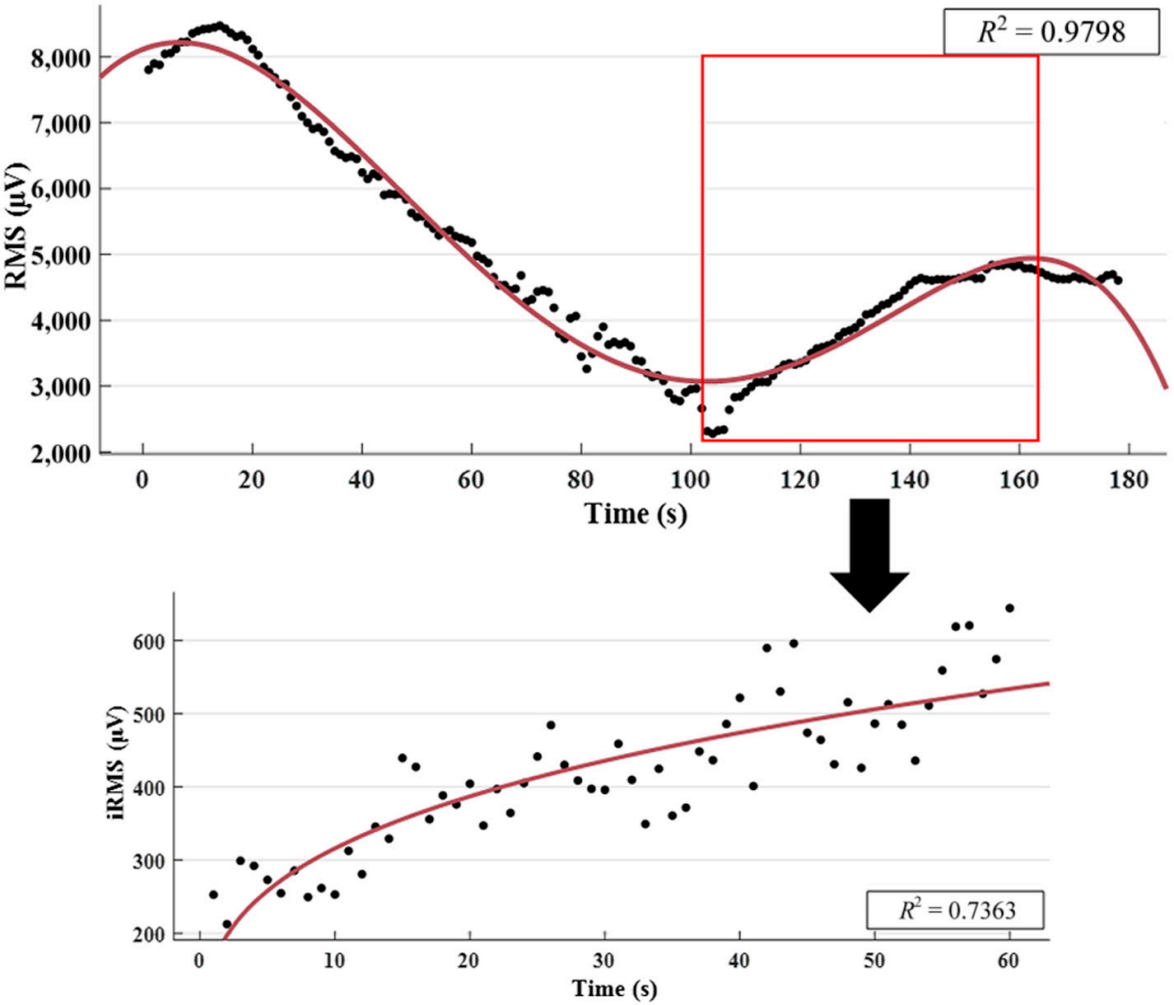
The corresponding relationship between the muscle response curve under FES and the fatigue characteristic curve under dumbbell weightlifting training.

Compared with the fatigue characteristic curve under dumbbell weightlifting training, as shown in Fig. 8, the curve is highly consistent with the third stage of the muscle response curve under FES. Therefore, the third stage can be used as the generation process of muscle fatigue, and the maximum value of the third stage curve is the sign that the muscle enters the fatigue state. After the muscle is subjected to FES experiment, the time to reach the maximum value is the maximum stimulation time of FES under the current.

### FES parameters’ effect on muscle

**Fig. 9.**
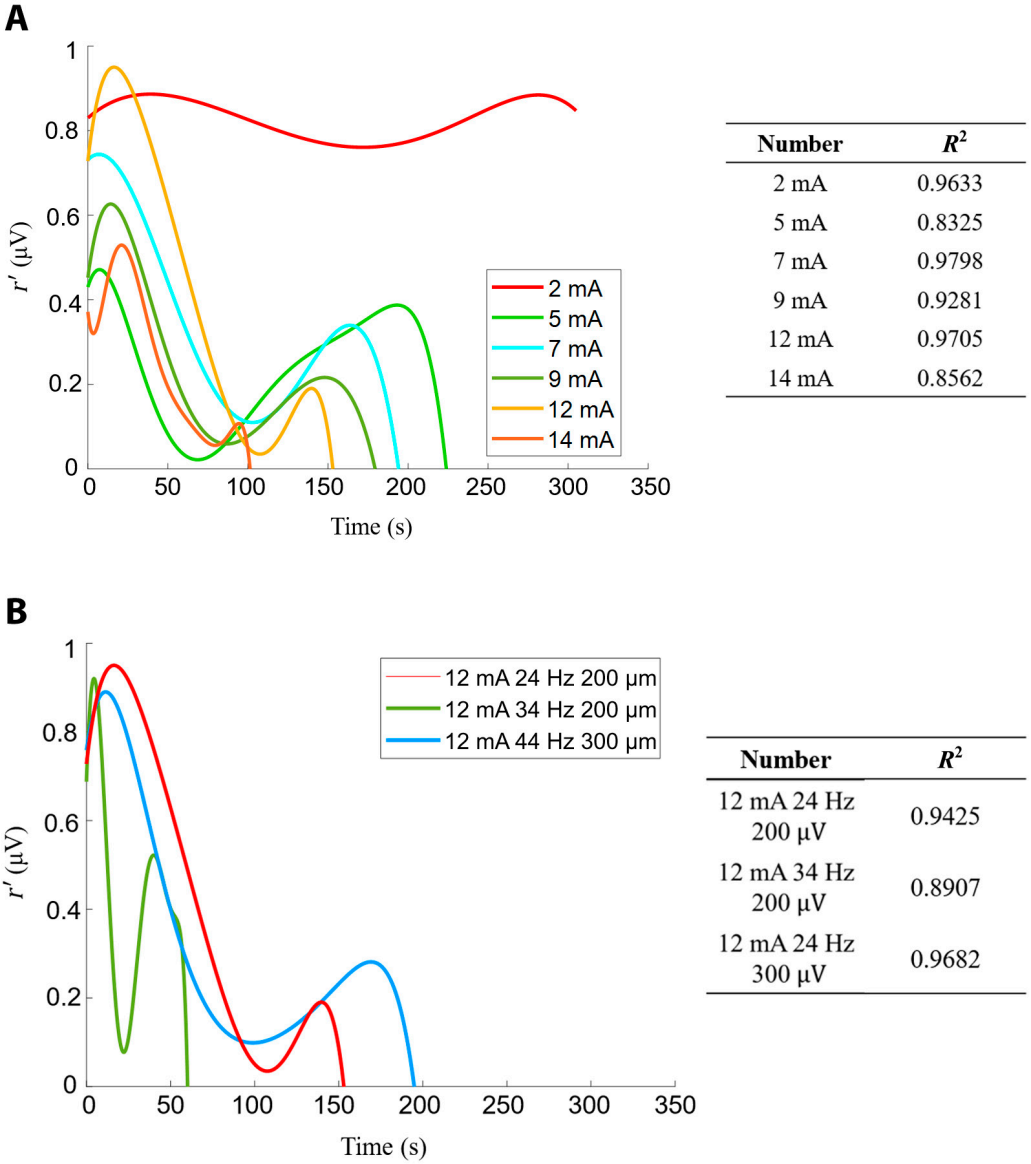
The fatigue characteristic curve under FES. (A) The fatigue characteristic curve under different current stimulations. (B) Current, frequency, or PW of the fatigue characteristic curve change results.

This part showed the FES parameters’ effect on the fatigue characteristic curve. The current amplitude effect on the fatigue characteristic curve is shown in Fig. 9A. It can be seen from the figure that when the current is increased, the maximum stimulation time will gradually become shorter, and this difference becomes more rapid as the current increases. The effect of current frequency and pulse width on the fatigue characteristic curve is shown in Fig. 9B. It can be seen from the figure that the maximum stimulation time is shortened when only the current frequency is increased. When only the current pulse width is increased, the maximum stimulation time is prolonged. Therefore, the increase in current will lead to the shortening of the maximum stimulation time, and there is a certain linear relationship curve. Low-frequency FES can prolong the stimulation time, while high-frequency FES can shorten the stimulation time. Increasing the pulse width of the current, the maximum stimulation time also increases, indicating that increasing the pulse width can prolong the stimulation time.

### Curve fitting results

**Fig. 10.**
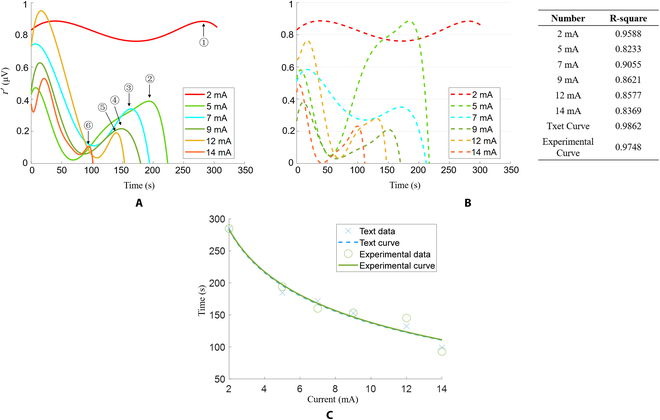
Fit results. (A) The maximum stimulation time under different current amplitudes (serial number pointing). (B) The test group’s muscle response curves under different currents. (C) The fitting relationship between current amplitude and fatigue generation time (experimental data and test data).

This part showed the linear relationship curve between the current amplitude and the maximum stimulation time. As shown in Fig. 10A, the maximum stimulation time under different current amplitudes is marked. By fitting the function of the current amplitude and the maximum stimulation time, the results represented by the solid line in Fig. 10C can be obtained. In addition, the fatigue characteristic curve under the same current amplitude is obtained in the cross-subject experiment, as shown in Fig. 10B. Although the difference in sEMG energy between the experimental group and the experimental group is relatively large, the difference in the maximum stimulation time was not large. The dashed line in Fig. 10C represents the test group’s fitting data. The results showed that the difference between the 2 curves under the same fitting is not large. Through this fitting relationship, it can be obtained that there is an inverse proportional relationship between the current size and the stimulation time, and the difference is statistically significant (*P* < 0.05); in other words, with the increase of the applied FES current, the faster the fatigue is generated, and the shorter the time it takes for the muscle to reach fatigue.

## Discussion

In this paper, the influence of FES parameters on the maximum stimulation time was explored, and the linear relationship between the current amplitude and the maximum stimulation time was finally obtained. The current frequency and pulse width are also the influencing factors of the maximum time. In order to get conclusive results, the fatigue characteristic curves under different fatigue levels were obtained through dumbbell weightlifting training. The establishment of this characteristic curve was achieved by analyzing the time domain characteristic RMS of the sEMG during the training process, and the different subjects’ muscle power difference effect was reduced by normalization. The power function fitting method better reflects the energy change of the muscle during the training process. The subjects’ muscle response curve under FES was obtained by the FES experiment, and the maximum stimulation time under different parameters was determined according to the characteristics of energy change during fatigue generation.

Some studies have shown that the sEMG induced by FES has “M” wave characteristics [31,32]. In their research, it was pointed out that FES can induce muscle factor activity, resulting in increased excitability of motor nerves, but with increasing stimulation, muscles gradually appeared fatigue, and the excitability of nerve fibers decreased [6]. This is consistent with the conclusion of this experiment. Differently, this paper discusses the causes of each section of the “M” wave in more detail, and refers to the fatigue generation process under autonomous motion, and finally determines the fatigue characteristic curve under electrical stimulation [33], which lays a foundation for a better understanding of the “M” wave, and also provides a scheme to avoid muscle damage for the parameter adjustment of FES technology.

Based on the fatigue characteristic curve under dumbbell weightlifting training, this paper explored the effect of FES on muscle fatigue and established a linear curve between the FES current amplitude and the maximum stimulation time. It was verified that the current frequency and pulse width also had an effect on the maximum stimulation time. In this paper, through the analysis of the “M” wave induced by FES, combined with the change of muscle energy under voluntary exercise, the time when the muscle reaches fatigue, in other words, the maximum stimulation time, was finally determined. This provides a way to use FES technology with adjustable parameters and no damage to the muscle. Through the linear curve model established in this paper, FES technology can play a better role, which makes it possible to further improve the use of FES technology.

## Data Availability

The data used to support the findings of this study are available from the corresponding author upon request.
